# Hypersensitive to Red and Blue 1 and Its Modification by Protein Phosphatase 7 Are Implicated in the Control of Arabidopsis Stomatal Aperture

**DOI:** 10.1371/journal.pgen.1002674

**Published:** 2012-05-10

**Authors:** Xiaodong Sun, Xiaojun Kang, Min Ni

**Affiliations:** Department of Plant Biology, University of Minnesota Twin Cities, Saint Paul, Minnesota, United States of America; Dartmouth College, United States of America

## Abstract

The stomatal pores are located on the plant leaf epidermis and regulate CO_2_ uptake for photosynthesis and the loss of water by transpiration. Their stomatal aperture therefore affects photosynthesis, water use efficiency, and agricultural crop yields. Blue light, one of the environmental signals that regulates the plant stomatal aperture, is perceived by the blue/UV-A light-absorbing cryptochromes and phototropins. The signal transduction cascades that link the perception of light to the stomatal opening response are still largely unknown. Here, we report two new players, Hypersensitive to Red and Blue 1 (HRB1) and Protein Phosphatase 7 (PP7), and their genetic and biochemical interactions in the control of stomatal aperture. Mutations in either *HRB1* or *PP7* lead to the misregulation of the stomatal aperture and reduce water loss under blue light. Both *HRB1* and *PP7* are expressed in the guard cells in response to a light-to-dark or dark-to-light transition. HRB1 interacts with PP7 through its N-terminal ZZ-type zinc finger motif and requires a functional PP7 for its stomatal opening response. HRB1 is phosphorylated *in vivo*, and PP7 can dephosphorylate HRB1. HRB1 is mostly dephosphorylated in a protein complex of 193 kDa in the dark, and blue light increases complex size to 285 kDa. In the *pp7* mutant, this size shift is impaired, and HRB1 is predominately phosphorylated. We propose that a modification of HRB1 by PP7 under blue light is essential to acquire a proper conformation or to bring in new components for the assembly of a functional HRB1 protein complex. Guard cells control stomatal opening in response to multiple environmental or biotic stimuli. This study may furnish strategies that allow plants to enjoy the advantages of both constitutive and ABA-induced protection under water-limiting conditions.

## Author Summary

Stomatal aperture is regulated by many environmental and biotic cues such as blue light, drought, elevated CO_2_ concentrations, high humidity, and pathogenic elicitors. Stomatal apertures vary over diurnal cycles, and stomata tend to be open during the day in response to blue light and tend to be closed at night. The blue/UV-A light-absorbing cryptochromes and phototropins are the receptors for the blue light response. We report the action of HRB1, a nuclear ZZ-type zinc finger protein, and PP7, a positive regulator of blue light signaling in the nucleus, in the signal transduction cascades downstream of blue light perception. Both *hrb1* and *pp7* mutants are more resistant to dehydration and show reductions in both water loss and blue light-regulated stomatal aperture. Our studies on their genetic and biochemical interactions offer novel insights on the network structure of the light signaling machinery and plant interactions with the environment. Periodic drought is one of the major environmental factors that limits biomass production and crop yield in a changing global climate. Our studies may open new possibilities to engineer plants to survive desiccation.

## Introduction

Phytochromes (phy) are photo-reversible red and far-red light receptors with five members in Arabidopsis, phyA to phyE [1]–[2]. The major red and far-red light responses include de-etiolation, photoperiodic flowering, and circadian rhythm. Cryptochromes (crys), including cry1 and cry2, are blue light-absorbing flavoproteins that regulate hypocotyl elongation, flowering time, circadian rhythm, and stomatal aperture [3]–[4]. Phototropins (phots), including phot1 and phot2, have a C-terminal serine/threonine kinase domain and repeated LOV1 (light, oxygen, or voltage-sensing domain 1) and LOV2 motifs in their N-terminus [5]–[6]. Phots regulate blue light-induced plant movements such as phototropism [5], chloroplast movement [6], and stomatal opening [7]. The phototropic and chloroplast movement responses allow plants to capture light energy efficiently or to avoid damage from high light intensity.

Guard cells control stomatal opening in response to many environmental or biotic stimuli such as blue light, drought, elevated CO_2_ levels, high humidity, and pathogens [8]–[9]. Stomata tend to be open during the day in response to blue light and to be closed at night over diurnal cycles [10]–[11]. In guard cells, phot1 and phot2 contribute equally to blue light-induced stomatal opening at fluence rates higher than 0.5 µmol/m^2^/s [7]. Similar to *phot1 phot2*, stomata of the *cry1 cry2* double mutant also show a reduced blue light response, whereas those of cry1-overexpressing plants show a hypersensitive response to blue light [12]. COP1 is a negative regulator of photomorphogenesis and directly interacts with either cry1 or cry2 [13]–[15]. Stomata of the *cop1* mutant are constitutively open in darkness [12]. RPT2 (ROOT PHOTOTROPISM2) contains an N-terminal BTB/POZ (broad complex, tramtrack, bric à brac/pox virus and zinc finger) domain and a C-terminal coiled-coil domain. RPT2 functions in the phot1-mediated stomatal opening response by interacting with phot1 *in vivo* [16].

Downstream of the photoreceptors, various intracellular signaling proteins are likely involved with multiple light responses and integrate signals of different light wavelengths. AtMYB60, an R2R3-MYB protein, is a positive regulator of stomatal aperture in response to blue light and diurnal cues. It is specifically expressed in the guard cells, and its expression is regulated by drought, crys, phyA, phyB, and COP1 [17]–[18]. A null *atmyb60* mutation results in a constitutive reduction of stomatal opening and reduced wilting under water stress. *AtMYB61* is another member of the *Arabidopsis R2R3-MYB* gene family and is also specifically expressed in guard cells [19]. Gain-of-function *AtMYB61* expression is both sufficient and necessary to cause reductions in stomatal aperture in response to light signals and diurnal cues.

The *hypersensitive to red and blue* 1 (*hrb1*) mutant has a short hypocotyl phenotype under red or blue light and a late flowering phenotype [20]–[21]. HRB1 is a small nuclear protein of 23 kDa with an unknown biochemical function [20], but its N-terminal ZZ-type zinc finger motif is likely involved in protein-protein interaction [22]. The *phosphatase 7* (*pp7*) knock-down plants have a long hypocotyl under blue light [23]. A loss-of-function *pp7* allele may be responsible for the light hypersensitivity in the *psi2* mutant, and PP7 interacts with nucleotide-diphosphate kinase 2 (NDPK2), a positive regulator of phytochrome signals [24]. PP7 has an intrinsic phosphatase activity although the substrates of PP7 remain largely unknown [25]. In this study, we report the involvement of both HRB1 and PP7 in the regulation of stomatal aperture under blue light and their genetic and biochemical interactions.

## Results

### HRB1 interacts with PP7 *in vitro*

**Figure 1:**
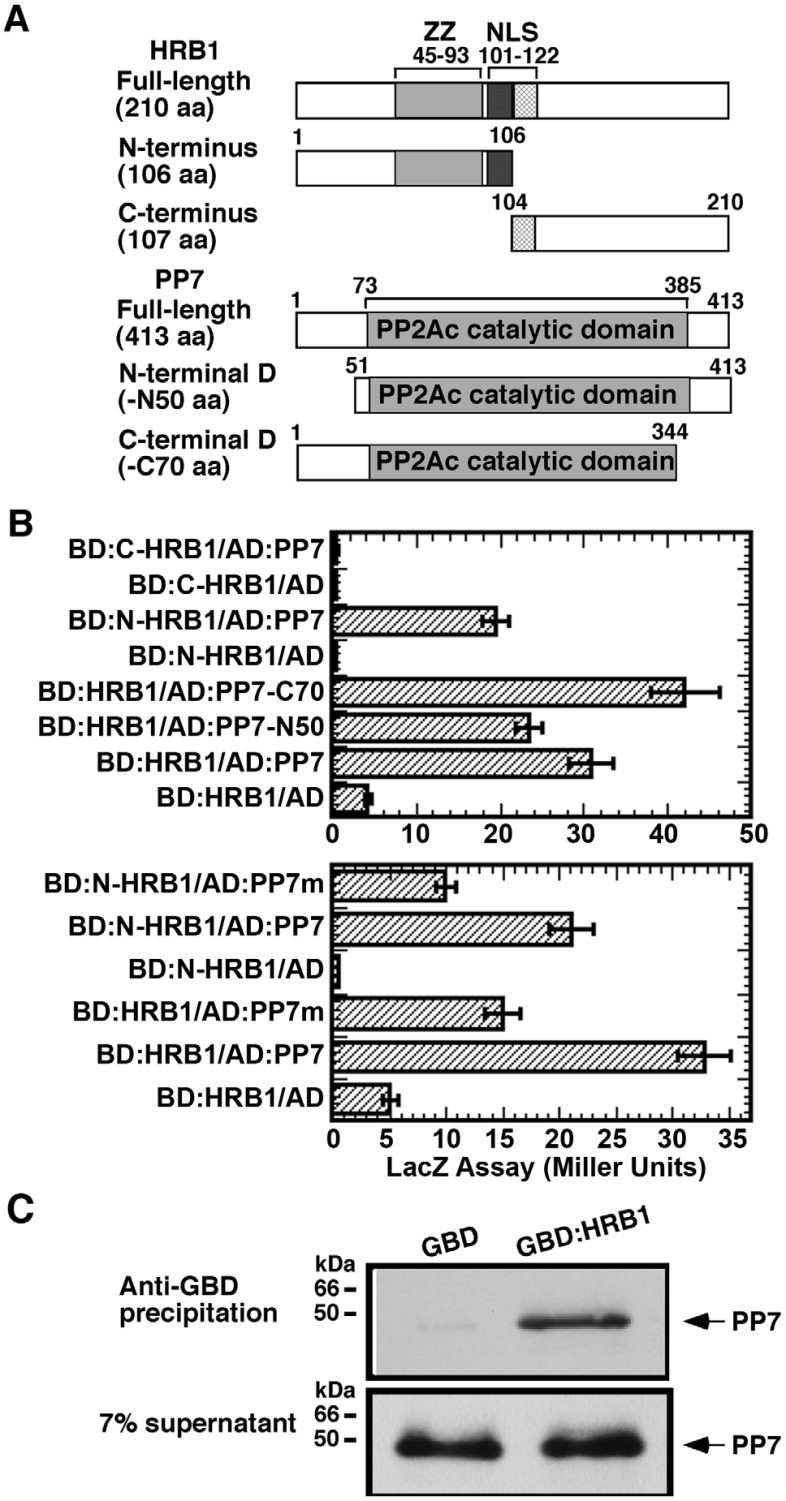
HRB1 interacts with PP7 *in vitro*. (A) Yeast two-hybrid bait and prey constructs. Full-length and truncated forms of the HRB1 bait proteins and PP7 prey proteins were fused to the GAL4 DNA binding domain (BD) and to the GAL4 activation domain (AD), respectively. (B) Yeast two-hybrid assay of the BD-HRB1 and AD-PP7 fusion proteins. PP7-N50 and PP7-C70 represent deletions in the N-terminus of 50 amino acids or in the C-terminus of 70 amino acids, respectively. N-HRB1 and C-HRB1 represent the N-terminus or C-terminus, respectively, of HRB1. PP7m contains a D to A change at amino acid position 116. (C) Pull-down assay using an anti-GBD antibody, GBD or GBD∶HRB1 bait produced in *E. coli* and PP7 prey produced in a TnT *in vitro* transcription and translation system. Autoradiography shows pelleted PP7 and 7% of the supernatant.

To explore the biochemical function of HRB1 in light signaling, a yeast two-hybrid library screen with HRB1 as bait was conducted, which identified PP7 as a potential HRB1-interacting protein. Because PP7 was identified as a blue light signaling component, we decided to further pursue this interaction. To map the interacting domain of HRB1, HRB1 was split into its N-terminal half and C-terminal half, each fused to the GAL4 DNA binding domain (Figure 1A). The N-terminal HRB1, most likely the ZZ-type zinc finger motif, interacted with full-length PP7 fused to the GAL4 activation domain in a quantitative yeast two-hybrid assay (Figure 1B, upper). The majority of the PP7 protein sequence is part of its catalytic domain, and the likely nature of this interaction may involve an enzyme-substrate relationship. Therefore, the first 50 amino acids at its N-terminus or the last 70 amino acids at its C-terminus were deleted, leaving its catalytic domain intact in both cases (Figure 1A). Both the full-length and the truncated PP7 interacted with HRB1, and the interaction between HRB1 and the PP7 C-terminal truncation was even stronger than that between HRB1 and full-length PP7 (Figure 1B, upper).

The interaction of HRB1 with a mutated version of PP7 that carries a D to A change at amino acid 116, a conserved residue in all protein serine/threonine phosphatases, was examined [27]. This aspartate residue in PP1 is critical for the conformation of its catalytic center, and a D to A change reduced its phosphatase activity 1000-fold [27]. The A116 mutation in PP7 reduced the interaction of PP7 with either the full-length or N-terminal HRB1 (Figure 1B, lower). Both wild type and mutated PP7 proteins accumulated at a similar level (Figure S1). Based on 3-dimensional structure and experimental data, D116 of PP1 is a critical residue in the catalytic core and interacts with a phosphorylated residue of the substrate [27]. A reduced yeast two-hybrid interaction of the mutated PP7 with the HRB1 N-terminus suggests that one of the phosphorylated residues is important for the interaction of PP7 with HRB1 and likely resides in the HRB1 N-terminus. This result also suggests that the catalytic and substrate recognition sites are closely linked in PP7. Subsequently, an *in vitro* immuno-precipitation (IP) assay was performed to verify the interaction of HRB1 with PP7 (Figure 1C). In this assay, the HRB1 protein, tagged with the GAL4 DNA binding (BD) domain and produced in *E. Coli*, interacted strongly with PP7, which was produced in an *in vitro* transcription-translation system and radiolabeled with ^35^S-methionine. In contrast, the BD domain alone did not significantly precipitate PP7 (Figure 1C).

### HRB1 interacts with PP7 *in vivo*

**Figure 2:**
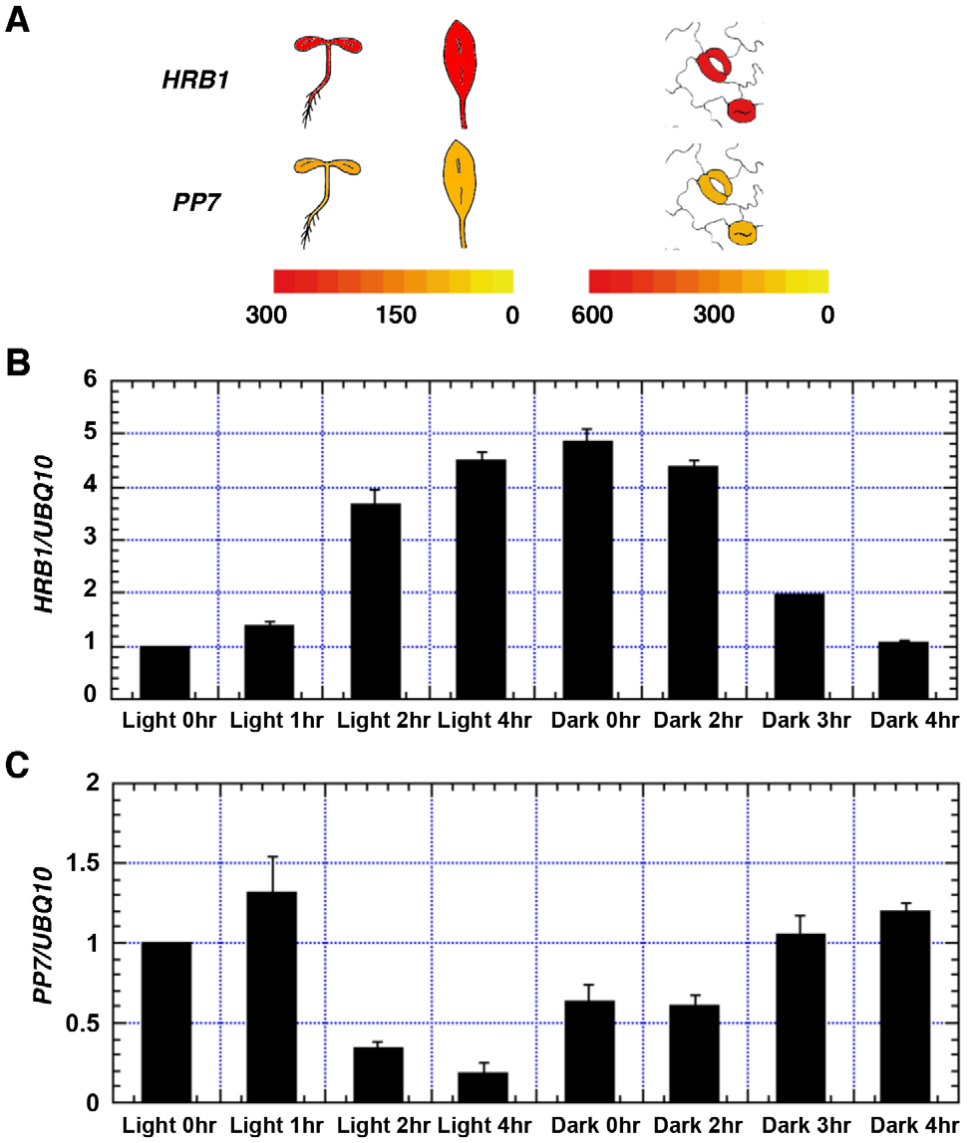
Both HRB1 and PP7 are expressed in guard cells. (A) Expression of *HRB1* and *PP7* in young seedlings and a rosette leaf (left color scale), and in a pair of guard cells (right color scale) acquired from the eFP browser. The color scales in the heat maps indicate normalized microarray expression values. Real-time RT-PCR analysis of the relative expression levels of *HRB1* (B) and *PP7* (C) in the transition from dark (light 0 hr) to white light for 1, 2 and 4 hours and in the transition from white light (dark 0 hr) to dark for 1, 2 and 4 hours. The Ws plants were grown in long days (16 hour light/8 hour dark) for 4 weeks under 40 µmol/m^2^/s white light.

**Figure 3:**
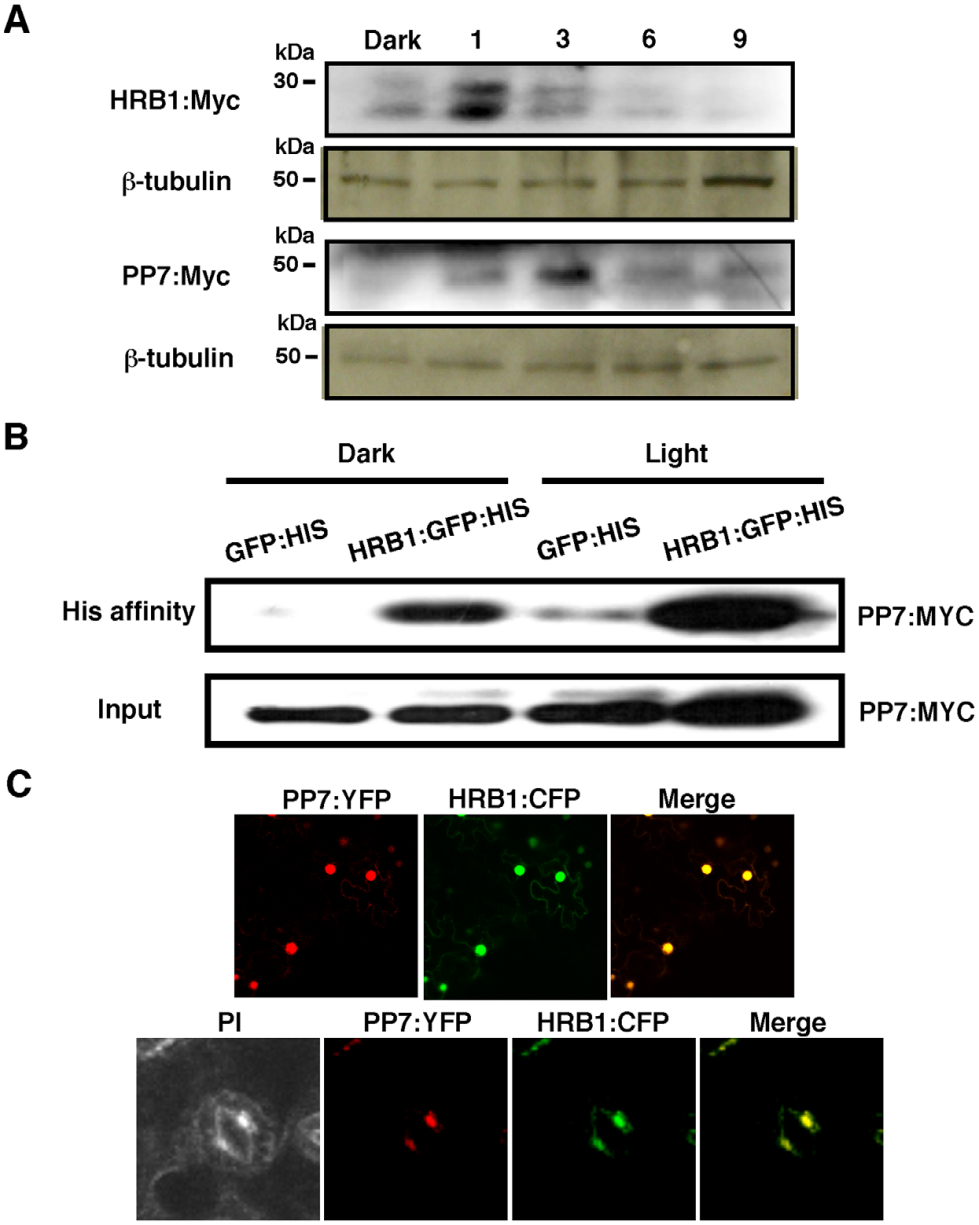
HRB1 interacts with PP7 *in vivo*. (A) Accumulation of HRB1∶Myc and PP7∶Myc as driven by the 35S CaMV promoter in the leaves of 4-week-old transgenic plants in the dark and in response to 4.93 µmol/m^2^/s blue light for 1, 3, 6 and 9 hours. The blots were stripped and re-probed with an antibody against ß-tubulin. (B) His affinity-precipitation of PP7∶Myc with GFP∶His or HRB1∶GFP∶His in the dark and under 4.93 µmol/m^2^/s blue light for 2 hours in Nicotiana leaves. (C) Co-localization of HRB1∶CFP and PP7∶YFP driven by the 35S CaMV promoter in Nicotiana leaf epidermal cells (upper panel) or driven by their native promoters in 2-week-old Arabidopsis leaf guard cells (lower panel) under 4.93 µmol/m^2^/s blue light for 2 hours. Propidium iodide (PI) fluorescence is shown in pseudo color to illustrate the cell shape.

For an interaction to occur, HRB1 and PP7 have to be transcribed and translated in the same cells at various stages of development. A search of the eFP gene expression database built on data from public microarray experiments [28] revealed that both *HRB1* and *PP7* are expressed in young seedlings, rosette leaves and guard cells (Figure 2A). *PP7* is predominantly expressed in guard cells in mature leaves [29]. *PP7* expression is also detected in hypocotyls at the young seedling stage and in the stems of mature plants [23]. The expression of both HRB1 and PP7 in guard cells was verified with their native promoters driving either an HRB1∶CFP or PP7∶YFP fusion (Figure 3C). In addition, expression of HRB1 was induced by light of various wavelengths [20]. Following a dark-to-light transition, the expression of *HRB1* was induced by light, and expression declined after a light-to-dark transition (Figure 2B). By contrast, the expression of *PP7* was suppressed by light and resumed following a light-to-dark transition (Figure 2C).

Because the abundance of both *HRB1* and *PP7* is influenced by light, the 35S promoter was used to drive the constitutive expression of *HRB1* and *PP7* to determine the levels of the HRB1 and PP7 proteins. The accumulation of the HRB1 protein was increased by a one-hour treatment with blue light and the accumulation of PP7 was also increased by blue light treatment for 3 hours (Figure 3A). The change in their protein levels indicates that both HRB1 and PP7 are post-translationally stabilized by blue light. Interestingly, HRB1∶myc migrated in two bands on an SDS-PAGE gel, and the two-band pattern was not altered in the dark or under blue light. The amount of either protein, however, gradually declined with prolonged blue light treatment up to 9 hours (Figure 3A).

The accumulation of HRB1 and PP7 is under stringent regulation by blue light (Figure 3A), and transgenic Arabidopsis plants may not accumulate sufficient HRB1 or PP7 protein for detection of their interaction by affinity-precipitation. An additional concern is that the interaction between an enzyme and a substrate may be transient or not very stable. However, the Nicotiana transient expression system allowed expression of sufficient HRB1 and PP7 for reproducible detection of their interaction. Based on the temporal accumulation of both proteins, an *in vivo* affinity-precipitation experiment was performed in the dark and after blue light treatment for 2 hours. HRB1∶GFP∶His but not GFP∶His precipitated Myc∶PP7 from plant extracts either in darkness or under blue light (Figure 3B), suggesting that their interaction does not require light. Next, a transient assay of HRB1∶CFP and PP7∶YFP in Nicotiana leaves showed that both proteins co-localized to the nucleus of epidermal cells (Figure 3C, upper panel). HRB1 and PP7 also co-localized in the nucleus of guard cells in stable transgenic Arabidopsis plants carrying HRB1∶CFP and PP7∶YFP (Figure 3C, lower panel). Thus, both proteins are normally expressed in the same cells, consistent with their interaction *in vivo*.

### HRB1 and PP7 interact to regulate stomatal aperture under blue light

**Figure 4:**
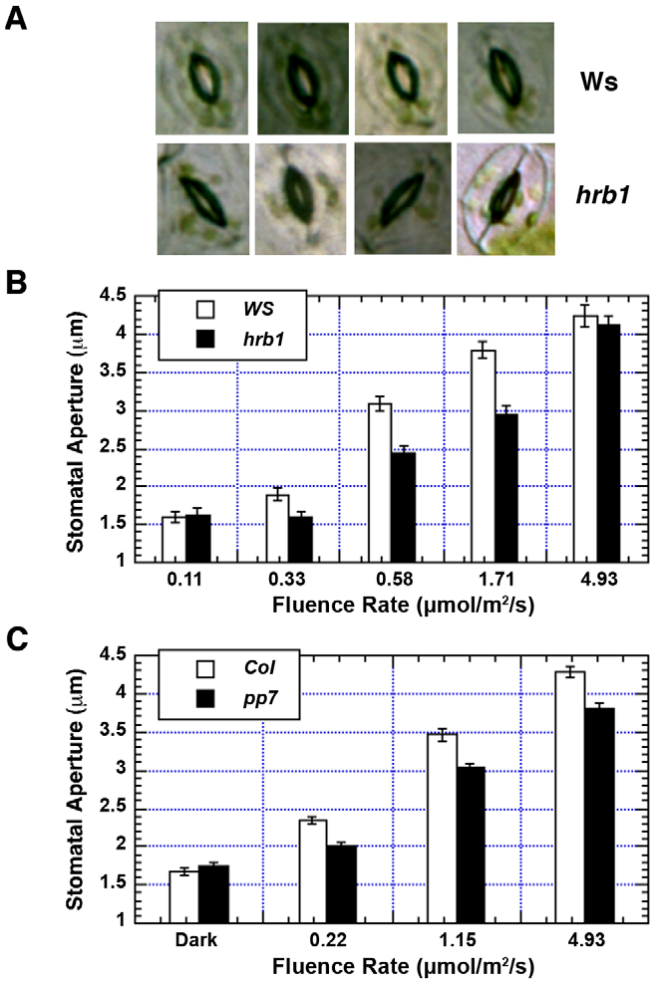
Both HRB1 and PP7 are involved in the regulation of stomatal aperture. (A) Stomatal aperture of 4-week-old Ws and *hrb1* plants under 2 µmol/m^2^/s blue light supplemented with 25 µmol/m^2^/s red light for 2 hours. (B) and (C) are the fluence responses of the stomatal opening in 4-week-old Ws and *hrb1* or 4-week-old Col and *pp7* plants under blue light supplemented with 25 µmol/m^2^/s red light for 2 hours. Data presented are means plus or minus standard errors (n = 70). The stomatal aperture of *hrb1* is significantly different from that of Ws at 0.33, 0.58, and 1.71 µmol/m^2^/s (P<0.0001, Student's two-tailed heteroscedastic t tests). The stomatal aperture of *pp7* is significantly different from that of Col at 0.22, 1.15, and 4.93 µmol/m^2^/s (P<0.001).

The *hrb1* mutant has a defective light response under both blue and red light (20). The stomatal opening response is regulated by blue light and enhanced by red light. *hrb1* had a much smaller stomatal aperture compared with wild type under weak to intermediate blue light (Figure 4A, 4B). The original studies on PP7 were performed in *PP7* knock-down lines, and we acquired SALK line 089764, which carries a T-DNA insertion in the second intron of the *PP7* gene from the Arabidopsis Biological Resource Center (23; Figure S2A). The T-DNA insertion was verified by PCR of genomic DNA prepared from wild type and this SALK line (Figure S2B). Reverse transcription PCR performed with two different primers before the T-DNA insertion failed to detect any *PP7* transcript in this line (Figure S2C). Consistent with the previous studies, this *pp7* knock-down line showed a long hypocotyl phenotype under blue light (Figure S1D). The *pp7* mutant also had a reduced stomatal aperture across a relatively broad range of blue light intensities compared with the Columbia (Col) wild type (Figure 4C).

**Figure 5:**
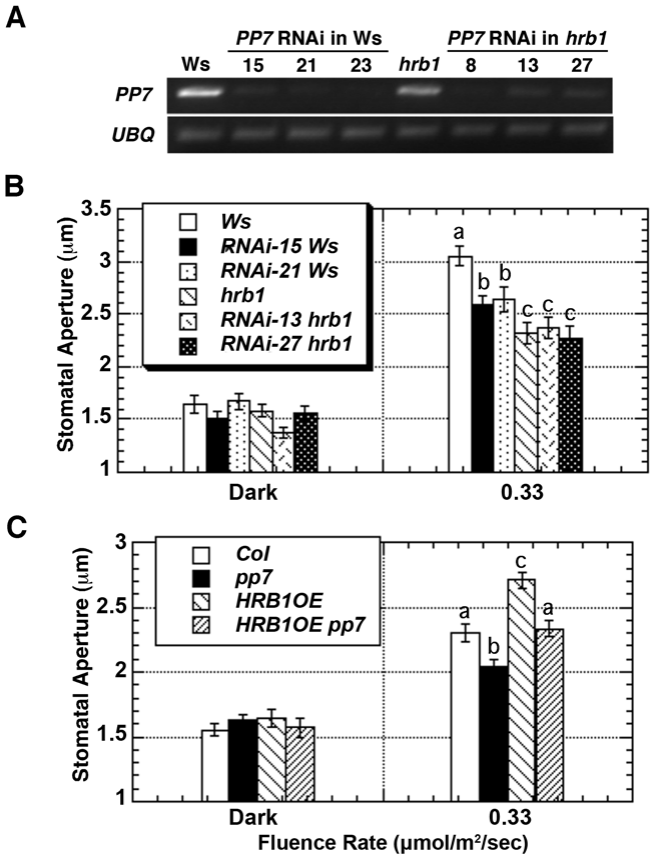
HRB1 and PP7 interact to regulate the stomatal aperture. (A) Semi-quantitative RT-PCR analysis shows the expression level of *PP7* in Ws, *hrb1*, and *PP7 RNAi* lines in either a Ws or *hrb1* background. (B) The stomatal aperture of 4-week-old Ws, *hrb1*, and *PP7 RNAi* lines in either an *hrb1* (*RNAi hrb1*) or Ws (*RNAi* Ws) background in the dark or treated with 0.33 µmol/m^2^/s blue light supplemented with 25 µmol/m^2^/s red light for 2 hours. Data presented are means plus or minus standard errors (n = 50). Significance levels: P<0.01 between a and b; P<0.0001 between a and c; P<0.05 between b and c. (C) Stomatal aperture of 4-week-old Col, *pp7*, and *35S::HRB1:MYC* in a Col (*HRB1OE*) or *pp7* (*HRB1OE pp7*) background. Significance levels: P<0.005 between a and b; P<0.005 between a and c; P<0.0001 between b and c. Plants were in the dark or treated with 0.33 µmol/m^2^/s blue light supplemented with 25 µmol/m^2^/s red light for 2 hours. Data presented are means plus or minus standard errors (n = 50).

The *hrb1* and *pp7* mutants are in different ecotypes, Wassilewskija (Ws) versus Col, and both genes are closely linked on chromosome 5. Thus, *PP7* RNAi lines were generated in the Ws and *hrb1* mutant backgrounds (Figure 5A). The stomatal opening response of the *hrb1 PP7* RNAi double mutant was similar to that of *hrb1* rather than additive, suggesting that HRB1 functions downstream of PP7 (Figure 5B). The hypocotyl phenotype of *hrb1* was also partially epistatic to that of the *PP7* RNAi lines (Figure S3). To better study their genetic interaction, HRB1 was over-expressed in the *pp7* mutant because *hrb1* and *pp7* have a very similar phenotype of stomatal aperture. Compared with Col, over-expression of *HRB1* increased stomatal aperture, and this phenotype was suppressed by the *pp7* mutation, suggesting that the action of the over-accumulated HRB1 requires functional PP7 (Figure 5C).

**Figure 6:**
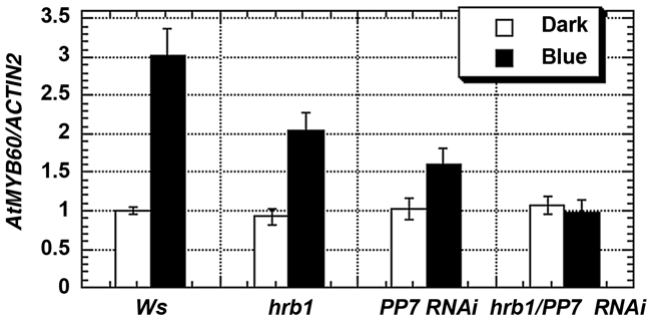
HRB1 and PP7 regulate the expression of *MYB60*. Quantitative RT-PCR analysis shows the expression levels of *MYB60* in Ws, *hrb1*, and *PP7 RNAi* lines in either a Ws (line 15) or *hrb1* (line 27) background in the dark or under 4.93 µmol/m^2^/s blue light for 3 hours. The expression of *MYB60* was examined with two biological samples and triplicate assays per biological sample. Data presented are means plus or minus standard errors.

To explore the downstream events regulated by HRB1 and PP7, the expression of *RPT2* [16], *AtMYB60* [17], *AtMYB61* [19], *ELF3* [30], and *FT* [30] was examined in the Ws, *hrb1*, the *PP7* RNAi lines, and the *hrb1 PP7* RNAi lines in the dark and under blue light (Figure 6). Blue light was selected because the *PP7* RNAi lines have a specific response under blue light [23]. We did not observe altered expression of *RPT2*, *AtMYB61*, *ELF3* and *FT* in either the *hrb1* or *PP7* RNAi lines. Although a misregulation of *FT* expression was observed in *hrb1* seedlings [20], the current experiments were performed with 4-week-old flowered plants grown under long days. As shown by others [17]–[18], the expression of *AtMYB60* was induced by blue light in Ws (Figure 6). The blue light-induced expression of *AtMYB60* was partially reduced by either the *hrb1* mutation or *PP7* RNAi and was blocked in the *hrb1 PP7* RNAi lines. Although the *hrb1 PP7* RNAi lines showed an *hrb1*-like stomatal phenotype, the *hrb1* mutation alone did not strongly suppress the expression of *AtMYB60* (Figure 5B and Figure 6), suggesting that HRB1 may target other genes in addition to *AtMYB60*.

**Figure 7:**
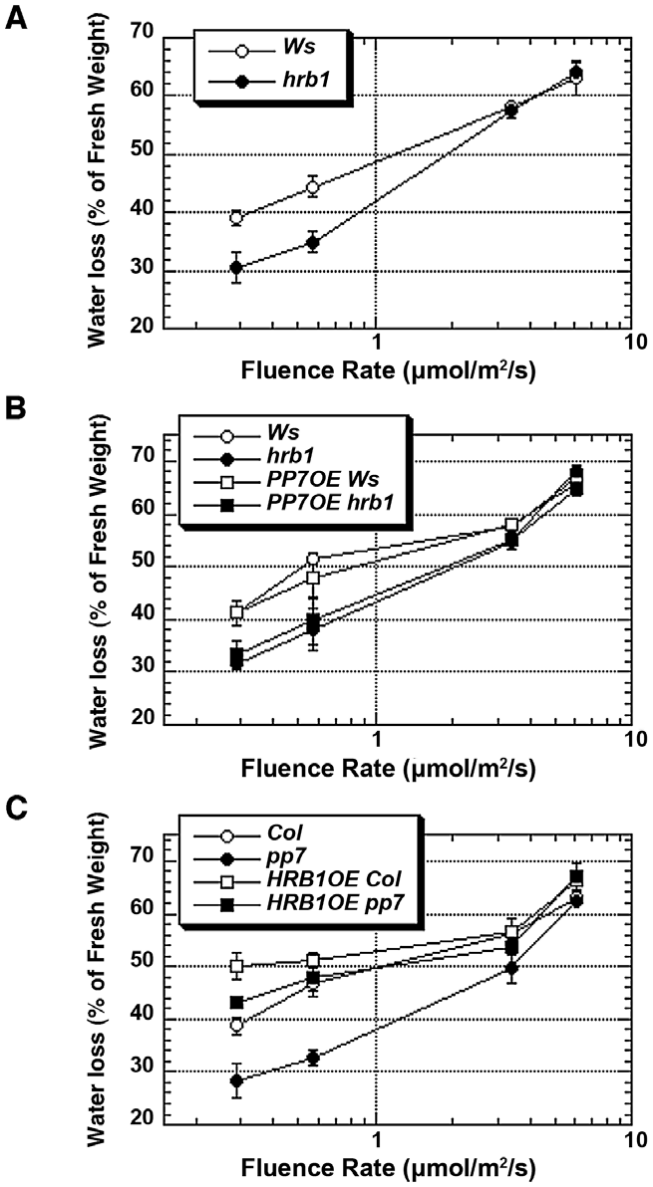
HRB1 action requires a functional PP7 during water loss. Water loss was measured in detached leaves from 4-week-old plants under blue light supplemented with 25 µmol/m^2^/s red light for 2 hours and calculated as the percentage of their initial fresh weight. (A) Water loss of *hrb1* plants is significantly different from that of Ws under 0.28 and 0.58 µmol/m^2^/s blue light (P<0.005, n = 10 and hereafter). (B) Water loss of *hrb1* or *35S::PP7:MYC* in *hrb1* (*PP7OE hrb1*) is significantly different from that of Ws or *35S::PP7:MYC* in Ws (*PP7OE Ws*) under 0.28 and 0.58 µmol/m^2^/s blue light (P<0.005). (C) Water loss of *pp7* is significantly different from that of Col under all blue light intensities except 6.06 µmol/m^2^/s (P<0.005). Water loss of *35S::HRB1:MYC* in Col (*HRB1OE Col*) is significantly different from that of Col under 0.28 and 0.58 µmol/m^2^/s blue light (P<0.005) and from that of *35S::HRB1:MYC* in *pp7* (*HRB1OE pp7*) under 0.28 µmol/m^2^/s blue light (P<0.05).

Detached leaves of the *hrb1* mutant lost less water under low to intermediate intensities of blue light compared with the Ws wild type (Figure 7A). The over-expression of *PP7* did not cause any visible phenotype in either the Ws or *hrb1* background, and the transgenic lines showed a very similar water-loss response to either the Ws or *hrb1* plants (Figure 7B). In contrast, the loss-of-function *pp7* mutant lost much less water compared with Col over a broad range of blue light intensities (Figure 7C). The over-expression of *HRB1* in the Col background promoted water loss, presumably due to a larger stomatal aperture. This promotion was partially suppressed by the *pp7* mutation (Figure 5C and Figure 7C), suggesting again that the action of over-accumulated HRB1 requires functional PP7.

### PP7 dephosphorylates HRB1

**Figure 8:**
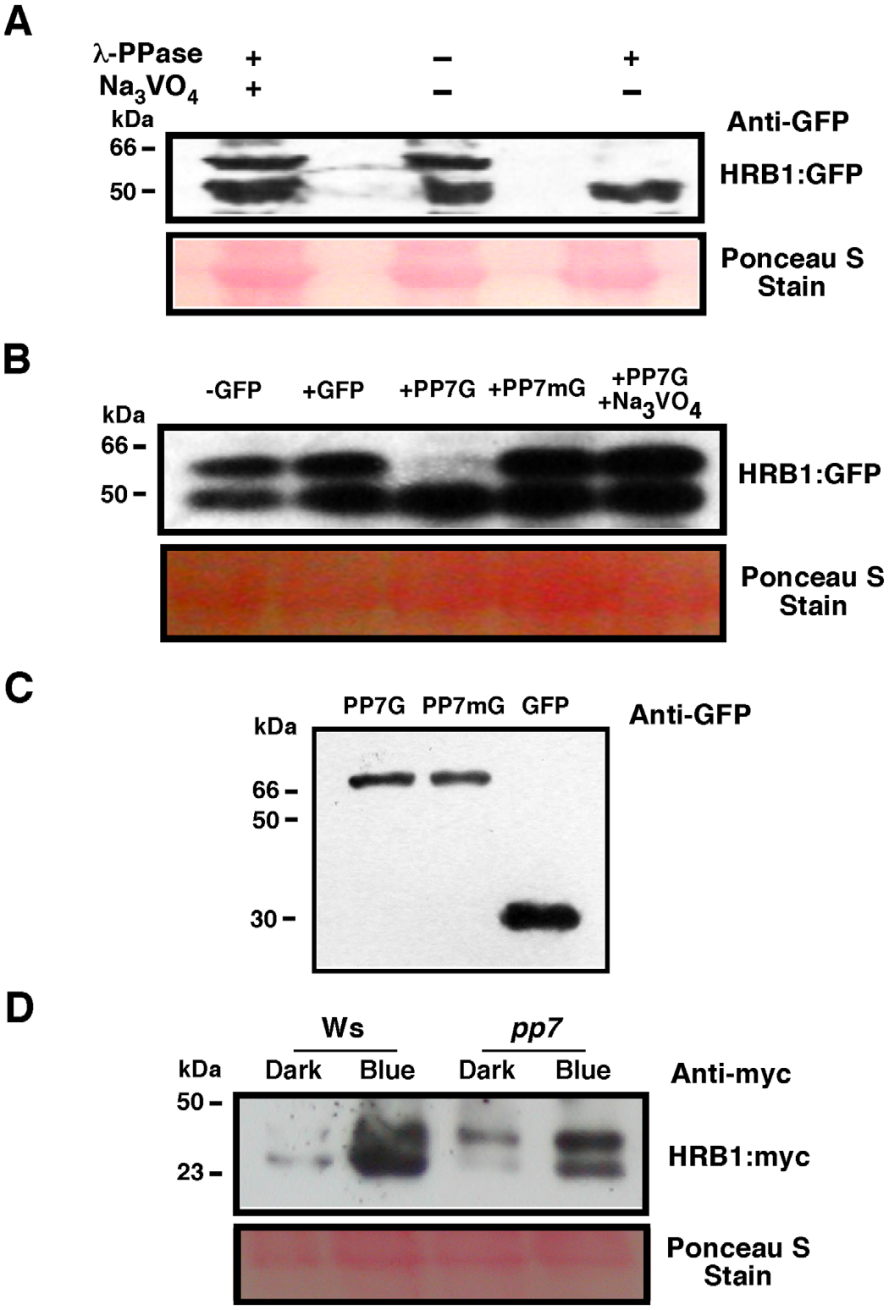
PP7 dephosphorylates HRB1. (A) Phosphorylation status of HRB1∶GFP from transgenic plants treated with or without lambda phosphatase or Na_3_VO_4_. (B) Phosphorylation status of HRB1∶GFP from transgenic plants treated with partially purified GFP, PP7∶GFP (PP7G), mutated PP7∶GFP (PP7mG), and PP7∶GFP (PP7G) in the presence of Na_3_VO_4_. (C) Gel showing the partially purified PP7G, PP7mG, and GFP proteins. (D) Phosphorylation status of HRB1∶Myc in the leaves of 3-week-old Col or *pp7* in the dark or under 4.93 µmol/m^2^/s blue light for 2 hours. Ponceau S staining revealed more proteins loaded in the Ws lane under blue light.

Western blot analysis reveals two bands of HRB1∶myc (Figure 3A). Treatment with lambda protein phosphatase eliminated the upper band, which was presumably the phosphorylated isoform of HRB1 (Figure 8A). The addition of Na_3_VO_4_, a phosphatase inhibitor, blocked the activity of the lambda phosphatase (Figure 8A). To determine whether PP7 can dephosphorylate HRB1, purified PP7∶GFP∶His and GFP∶His fusion proteins from plant extracts were mixed with total protein extracts prepared from HRB1∶GFP transgenic Arabidopsis plants. The addition of PP7 depleted phosphorylated HRB1∶GFP protein (Figure 8B), and this activity was inhibited by Na_3_VO_4_. PP7 carrying a D to A mutation at position 116, a conserved residue in all protein serine/threonine phosphatases [25], failed to deplete phosphorylated HRB1 (Figure 8B). Curiously, the level of the lower dephosphorylated HRB1 band showed no concomitant increase when the intensity of the upper phosphorylated HRB1 band decreased. Although the total HRB1 should be the sum of the modified and unmodified forms, we noticed that the dephosphorylated HRB1 was relatively unstable at 30°C for 30 minutes, suggesting that dephosphorylated HRB1 may be more susceptible to degradation.

To determine if PP7 dephosphorylates HRB1 *in vivo*, the phosphorylation status of HRB1∶Myc was examined in the leaves of 3-week-old Col or *pp7* mutant in the dark or after illumination with 4.93 µmol/m^2^/s blue light for 2 hours. Gel filtration analysis was performed on protein extracts and the resulting protein blots were probed with an antibody against Myc (Figure 8D). In Ws, HRB1∶Myc mostly existed in its dephosphorylated form in the dark or under blue light. In contrast, more phosphorylated HRB1∶Myc accumulated in *pp7* in the dark or under blue light. A substantial fraction of HRB1∶Myc, however, is still dephosphorylated in *pp7* under blue light. This portion may represent free HRB1∶Myc which may be more accessible to action by non-specific phosphatases released during the isolation process than HRB1∶Myc in a protein complex. Alternatively, at least some of the effects of PP7 on the HRB1 phosphorylation status in PP7 wild type versus pp7 mutants could be indirect.

### PP7 activity is required to assemble a functional HRB1 protein complex

**Figure 9:**
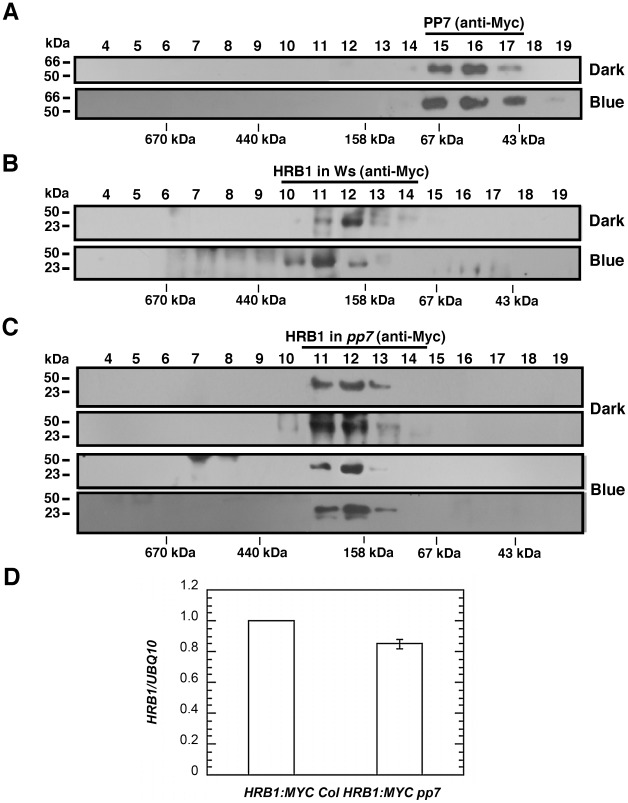
PP7 affects the assembly of a functional HRB1 protein complex. (A) Gel filtration profile of PP7∶Myc from the leaves of 4-week-old Col transgenic plants in the dark (top) or under 4.93 µmol/m^2^/s blue light for 3 hours (bottom). Gel filtration profile of HRB1∶Myc from the leaves of 4-week-old Col (B) or *pp7* (C) plants in the dark (top) or under 4.93 µmol/m^2^/s blue light for 3 hours (bottom). (D) Real-time RT-PCR analysis shows the relative expression of *HRB1∶MYC* in a Col (*HRB1∶MYC* Col) or *pp7* (*HRB1∶MYC pp7*) background.

The *pp7* mutation did not affect the nuclear localization of the HRB1∶GFP protein (Figure S4). The level of either the *HRB1∶Myc* message or HRB1∶Myc protein accumulation in 35S::HRB1:Myc transgenic plants was not significantly altered in the *pp7* mutant background (Figure 9B–9D). Gel filtration experiments were performed with either HRB1∶GFP and PP7∶Myc expressed in a single plant or HRB1∶Myc and PP7∶Myc expressed in separate plants. The two approaches produced similar results. To estimate the true molecular mass of the protein complex, plants expressing either HRB1∶Myc or PP7∶Myc were used. In darkness or under blue light, the PP7∶Myc protein was detected in gel filtration peak fraction 16, with a molecular mass of its monomer or larger (Figure 9A). In contrast, the HRB1∶Myc protein was detected in peak fraction 12, at a size of 193 kDa, in the dark (Figure 9B). After blue or blue plus red light treatment for 2 hours, the peak of the HRB1∶Myc protein complex shifted to fraction 11, with a molecular mass of 285 kDa (Figure 9B). Occasionally, HRB1∶Myc could be detected in various fractions of smaller molecular mass, ranging from the size of its monomer to larger than its monomer. The pattern of the blue light-induced size shift of the HRB1 protein complex remained the same in either *cry1 cry2* or *phot1 phot2* double mutant, suggesting that either pair of blue light receptors is required for this light-induced response (Figure S5A and S5B). The light-induced size shift of the HRB1 protein complex was, however, compromised in the *pp7* mutant (Figure 9C). First, the peak of the HRB1 protein complex remained in fraction 12 both in the dark and under blue light in the *pp7* mutant (Figure 9C). Second, HRB1∶Myc was predominately phosphorylated in the *pp7* mutant plants both in the dark and under blue light (Figure 9C).

## Discussion

### Both HRB1 and PP7 control the stomatal aperture response

Blue light is one of the major environmental cues to regulate stomatal aperture with phots and crys being the responsible photoreceptors for this response. Both *hrb1* and *pp7* were isolated as hypocotyl mutants under red and blue light or blue light alone [20], [24]. We report their defects in the light-induced stomatal opening response in this study (Figure 4). Compared with *hrb1*, *pp7* has a relatively weak stomatal aperture phenotype but a stronger phenotype in water retention, probably due to the difference between ecotypes, Col versus Ws (Figure 4C and Figure 7C). There was an altered phototropic response for *hrb1* compared with Ws but not for *pp7* compared with Col (Figure S6).

Both HRB1 and PP7 positively regulate the stomatal opening response but play opposing roles in hypocotyl elongation. Unlike PP7, HRB1 negatively regulates hypocotyl elongation under blue light. The phosphorylation status of HRB1 may have a different effect on the two responses or the action of HRB1, and its modification by PP7 may activate different downstream events in hypocotyl cells and guard cells. A dual but opposite function has also been observed for EARLY FLOWERING 3 (ELF3) in the regulation of different light responses. A loss-of-function *ELF3* allele was identified as *scs1-1* (suppressor of closed-stomata phenotype in *phot1 phot2*) in Arabidopsis [30]. All *elf3* loss-of-function alleles show wide-open stomata either in the dark or under blue light. Thus, ELF3 negatively regulates the phototropin-mediated stomatal opening but positively regulates hypocotyl elongation under red light [30], [31].

Both the *phot1 phot2* and *cry1 cry2* double mutants showed a reduced stomatal aperture phenotype, and the two pairs of photoreceptors function independently [12]. In our current studies, the light-induced size shift of the HRB1 protein complex is not altered in either the *phot1 phot2* or *cry1 cry2* double mutant, suggesting that HRB1 and PP7 function in both the phot and cry signaling pathways (Figure S5A and S5B). We overexpressed HRB1 in either the *phot1 phot2* or *cry1 cry2* double mutant and the transgenic plants showed a stomatal phenotype similar to either of the two double mutants (Figure S5C). In contrast, HRB1 overexpression in the background of wild type crys and phots did cause a stomatal aperture phenotype (Figure 5C). Why the overexpression of HRB1 in the *cry1 cry2* double mutant with a wild type phot1 phot2 background failed to generate a stomatal aperture phenotype and vice versa if HRB1 functions in either pathway is intriguing. One likely reason is that the overexpression of HRB1 in the phot pathway may not be strong enough to override the *cry1 cry2* mutant phenotype.

### HRB1 physically interacts with PP7

HRB1 and PP7 were demonstrated to interact in a yeast two-hybrid system, *in vitro*, and *in vivo* (Figure 1 and Figure 3). PP7 belongs to a large family of serine/threonine protein phosphatases in Arabidopsis. This family is divided into seven clusters, PP1 to PP7, based on the amino acid sequences of their catalytic subunits [32]. PP1 has been reported as a positive regulator in blue light-mediated stomatal opening, acting downstream of phototropins but upstream of the H^+^-ATPase [33]. PP7 is a relatively large protein with the majority of its sequence in its catalytic domain. The N-terminal ZZ-type zinc finger motif of HRB1 and the catalytic domain of PP7 mediate their interaction (Figure 1B). The hypothesis whether HRB1 regulates the activity of PP7 through an *in vitro* phosphatase activity assay was then tested. The addition of recombinant HRB1 protein did not alter the phosphatase activity of PP7 toward its synthetic peptide substrate, suggesting that HRB1 may not act upstream of PP7.

### PP7 dephosphorylates HRB1

HRB1 migrates as two bands on an SDS-PAGE gel, consistent with posttranslational modifications such as phosphorylation or ubiquitination. Either lambda phosphatase or PP7 treatment eliminated the upper band of HRB1∶GFP, i.e. the phosphorylated form of HRB1 (Figure 8A and 8B). Full-length PP7 protein produced in *E. coli* tends to form inclusion bodies and requires complicated denature-refolding treatment to recover its phosphatase activity [25]. A truncated form of PP7 with part of its catalytic domain deleted is easily purified and has stronger activity toward an artificial substrate [25]. However, this truncated form of PP7 did not dephosphorylate HRB1 efficiently. Instead, we partially purified PP7∶GFP∶His from transgenic plants and retained its activity toward HRB1 in a similar way used by others [34].

HRB1 belongs to the Drought-Induced 19 (Di19) protein family [26]. Several Di19 family proteins were phosphorylated by the calcium-dependent protein kinases CPK4 and CPK11 to regulate ABA-mediated stomatal aperture responses [26], [35]–[36]. NetPhos software predicted 10 serine and 3 threonine sites as potential phosphorylation sites of Di19 [37]. Studies with mass spectrometry have identified at least two phosphorylation sites, Thr105 and Ser107, in Di19 [35]. Thr105 but not Ser107 is conserved in HRB1, and HRB1 has likely acquired additional phosphorylation sites. Phosphorylation at the Ser-Pro and Thr-Pro sites also changes the electrophoretic mobility of a protein [38]. Two Ser-Pro sites were identified in HRB1 by the NetPhos program, and these sites may be involved in the phosphorylation and dephosphorylation of HRB1. In vivo identification of the true phosphorylation sites remains challenging and will be the focus of our future studies.

### PP7 activity is important for the formation of HRB1 protein complex

HRB1 exists in a protein complex mostly in its dephosphorylated form, and the size of this complex is approximately 193 kDa in the dark. Blue light caused a size increase of the protein complex but did not alter the phosphorylation status of HRB1 (Figure 9B). The phosphorylation status of HRB1 was not altered in the wild type under various light wavelengths (Figure S7). In addition, there was no alteration of HRB1 phosphorylation status in the *phyA* mutant under far-red light, in the *phyB* mutant under red light, and in the *cry1 cry2* or *phot1 phot2* double mutants under blue light. This light-induced size shift was not altered in either the *phot1 phot2* or *cry1 cry2* double mutant, and both pairs of photoreceptors appear to mediate this response (Figure S5A and S5B). Attempts to introduce HRB1∶Myc to a quadruple *cry phot* mutant have been unsuccessful because the quadruple mutant was weak and difficult to transform.

Crys are mainly involved in the regulation of gene expression in the nuclei, and phots are localized in the plasma membrane. The current data appear to argue that crys may be the photoreceptors or crys may closely associate with the function of the nuclear HRB1 and PP7 proteins. Even if crys and phots employ different signal transduction pathways to regulate stomatal opening, the pathways eventually merge. In the case of HRB1, blue light may regulate another component that is shared by two signaling pathways after their merger. This component may be involved in the observed size shift of the HRB1 protein complex under blue light. Alternatively, whether phot signaling requires nuclear events for a sustained response remains an open question. For example, COP1 is a predominantly nuclear protein and also functions downstream of phot1 and phot2 [4]. Phot2 has been recently demonstrated to promote palisade cell development [39].

The size increase of the HRB1 complex induced by blue light was compromised in the *pp7* mutant, suggesting that the proper modification of HRB1 is required to bring in new components to the protein complex (Figure 9C). Alternatively, HRB1 in the protein complex may adopt a different conformation upon dephosphorylation by PP7. PP7 migrated as a monomer or slightly larger (Figure 9A). Occasionally, HRB1 was also detected in smaller molecular weight fractions in either its phosphorylated or dephosphorylated form. The gel filtration fractions may contain interacting HRB1 and PP7 and/or a partially assembled HRB1 protein complex. Interestingly, a fraction of HRB1 was still dephosphorylated in the *pp7* mutant. Other protein phosphatases may potentially dephosphorylate HRB1 at various sites to shape the final mobility pattern of the HRB1 protein on an SDS-PAGE gel, especially when cell structures were ruptured during the protein isolation process.

## Materials and Methods

### Plant growth and light conditions

The Arabidopsis thaliana ecotype Wassilewskija and Columbia were used as wild types. Monochromatic red, far-red, or blue light was generated with an LED SNAP-LITE (Quantum Devices, Barnereld, WI). Light intensity and peak wavelength were measured with a SPEC-UV/PAR spectroradiometer (Apogee Instruments, Logan, UT).

### Yeast two-hybrid screen and ß-galactosidase assay

The yeast two-hybrid library screen was conducted as described previously with a Matchmaker GAL4 two-hybrid system 3 [Clontech, Mountain View, CA, 40]. The baits in the pGBKT7 vector and the preys in the pGADT7 vector were introduced into the yeast stains by the PEG transformation method (Yeast Protocols Handbook by Clontech, Mountain View, CA). Transformants were selected on minimal synthetic dropout (SD) medium lacking Trp and Leu, and the ß-galactosidase activity assay was performed as described (Yeast Protocols Handbook by Clontech, Mountain View, CA). Total yeast protein was isolated as described (Yeast Protocols Handbook by Clontech, Mountain View, CA), and the western blot was probed with an anti-HA antibody (GenScript, Piscataway, NJ).

### *In vitro* immuno-precipitation assay

PCR fragments containing the HRB1 cDNA and GAL4 BD sequences were subcloned into the pRSETB vector and transformed into *E. Coli*. Total protein extract was prepared from *E. Coli* cells in pull-down buffer (1× PBS buffer, pH 7.5, 0.1% NP40, and a full set of proteinase inhibitors). Approximately 50 µg of total *E. Coli* protein extract was mixed with 2.5 µl anti-GAL4 BD antibody (Santa Cruz Biotechnology, Santa Cruz, CA), 10 µl protein A/G plus agarose beads (Santa Cruz Biotechnology, Santa Cruz, CA) in 500 µl cold pull-down buffer plus 0.05% BSA. The mixture was incubated at 4°C for 2 hours, washed 3 times with 500 µl cold pull-down buffer minus BSA, and incubated with 4 µl of in vitro translated PP7 at 4°C for 2 hours. For *in vitro* translation, 40 µl TNT *in vitro* translation master mix (Promega, Madison, WI) was mixed with 1 µg pRSETB-PP7 DNA and 2 µl ^35^S-labeled methionine (MP Biomedicals, Santa Ana, CA) and incubated at 30°C for 1 hour. The *in vitro* binding mixture was washed 3 times with cold pull-down buffer, added to 4 µl 5× SDS loading buffer, boiled for 3 minutes, and loaded onto a 12% SDS-PAGE gel. After electrophoresis, the gel was air-dried and exposed to BioMax MS film (Kodak, Rochester, NY) with an intensifying screen at −80°C.

### Plant protein extraction and Western blots

Plant tissues were frozen in liquid nitrogen and ground in plant protein extraction buffer (50 mM Tris-Cl, pH 7.5, 150 mM NaCl, 10% glycerol, 1% Triton X-100, and a full set of proteinase inhibitors) at a ratio of 0.5 ml per gram fresh weight. The extracts were centrifuged at 20,000 g at 4°C for 30 minutes, and the supernatant was recovered. Approximately 80 µg of total protein was loaded to a 12% SDS-PAGE gel and blotted onto an Immobilon P membrane. The membrane blots were probed with anti-GFP or anti-Myc primary antibodies (Santa Cruz Biotechnology, Santa Cruz, CA) and an anti-mouse secondary antibody (Sigma-Aldrich, St. Louis, MO). The blots were also stripped and re-probed with a primary antibody against ß-tubulin (Santa Cruz Biotechnology, Santa Cruz, CA).

### *In vivo* affinity-precipitation and co-localization

PCR-amplified HRB1 genomic DNA and PP7 cDNA were cloned into pCR8/GW/TOPO vectors, and then recombined into pMDC83 (HRB1∶GFP∶His) and pMDC203 (PP7∶Myc) vectors, respectively [41]. The forward and reverse primers for HRB1 were ATGGATTCGAATTCATGG and TCCCCCCGGGAACTTGTCTTCAAGCATGG, and for PP7, were ATGGAAACTGTTCCACCA and GCTATTTGGTTGTTCGTT.

An overnight agrobacterium culture was diluted 1∶40 in LB medium and grown at 30°C for 16 hours. Cells were collected after centrifugation at 8,000 rpm for 2 minutes, resuspended in MES buffer (10 mM MES, pH 5.6, 10 mM MgCl_2_, and 150 µM Acetosyringone), and incubated at 30°C with gentle shaking for 2 hours. Agrobacteria carrying the pMDC83-control or pMDC83-HRB1 vector, pMDC203-PP7 vector, and pBin61-P19 at a ratio of 1∶1∶1 were co-infiltrated into the leaves from well-watered 6-week-old nicotiana benthamiana plants. P19 encodes a suppressor of gene silencing and thus significantly increases the amount of protein produced in this transient expression system [42]. Leaf tissues were harvested 3 (without P19 co-infiltration) or 5 days (with P19 co-infiltration) after the initial infiltration [43].

Approximately 12 g of Nicotiana leaves was frozen in liquid nitrogen and ground in 10 ml of cold co-precipitation buffer (50 mM Tris-Cl, pH 7.5, 150 mM NaCl, 10% glycerol, 0.5% NP40, 10 mM imidazole, and a full set of proteinase inhibitors). After centrifugation at 20,000 g and 4°C for 30 minutes, the supernatant was incubated with 200 µl pre-equilibrium nickel-agarose beads (Qiagen, Valencia, CA) at 4°C for 2 hours [34]. The incubation mixture was then washed 5 times with cold co-precipitation buffer, and the proteins attached to the column were eluted with 100 µl cold co-precipitation buffer containing 200 mM imidazole. Approximately 50 µl of the elution was mixed with 10 µl 5× SDS loading buffer and loaded onto an SDS-PAGE gel. After blotting onto a membrane, PP7 was detected with an anti-Myc antibody (Santa Cruz Biotechnology, Santa Cruz, CA).

The HRB1 and PP7 genomic DNA in the pCR8/GW/TOPO vectors were also recombined into the pEarleyGate102 (HRB1∶CFP) and pEarleyGate104 (PP7∶YFP) vectors, respectively [44]. Agrobacteria carrying the pEarleyGate102 and pEarleyGate104 vectors were co-infiltrated into Nicotiana leaves. The pEarleyGate102-HRB1∶CFP and pEarleyGate104-PP7∶YFP or pMDC83-HRB1∶GFP∶His constructs were also introduced to Arabidopsis by the Agrobacterium-mediated vacuum infiltration method [45]. Infiltrated Nicotiana plants or 2-week-old transgenic Arabidopsis plants were kept in darkness for 3 days and treated with or without blue light for 2 hours. The leaves of the plants were soaked in 95% ethanol at 30°C for 1 hour and stained with propidium iodide (PI) for 10 minutes before images were taken with a Nikon C1si Laser Scanning Confocal Microscope equipped with a three-channel PMT detector. The exciting wavelengths for CFP, YFP, PI, and GFP were 458, 514, 561 and 488 nm, respectively.

### Stomatal aperture and water loss measurements

The stomatal aperture was measured according to Mao et al. [12]. Arabidopsis plants that were 3 to 4 weeks-old were grown in darkness for 72 hours, and their epidermal layers were attached to adhesive tape and peeled off from the abaxial side of the leaf under dim green light. The epidermal strips were then floated in 10 ml of basal reaction buffer (5 mM MES, pH 6.5, 50 mM KCl, and 0.1 mM CaCl_2_) and kept in the dark for 1 hour. The epidermal strips were subsequently illuminated with blue light supplemented with 25 µmol/m^2^/s red light for 2 hours. Images were acquired with an Olympus BX53 fluorescent microscope with a Spot Insight 4 MP CCD camera and analyzed using ImageJ software.

For the water loss experiments, Arabidopsis leaves were detached and kept under blue light plus 25 µmol/m^2^/s red light at 30% humidity. The detached leaves were weighed every 30 minutes and the rate of water loss was calculated as the percentage of their initial fresh weight [12], [46].

### PP7 RNA interference

A 365-bp DNA fragment from the PP7 cDNA sequence was PCR-amplified, cloned into the pCR8/GW/TOPO vector, and recombined into the pAGRIKOLA vector [47]. The forward and reverse primers were CACCACCGTCGGGTAGTTCTTCT and GCATCTGGACCTTCATGT. This construct was transformed into Arabidopsis by the Agrobacterium-mediated vacuum infiltration method [45].

### Phosphatase assay

Total protein was extracted from 0.5 g pMDC83-HRB1∶GFP∶His transgenic plants in 200 µl cold 2× PPase reaction buffer (1× buffer contains 50 mM HEPES, pH 7.5, 100 mM NaCl, 10% glycerol, 0.5% Triton X-100, and a full set of proteinase inhibitors). Approximately 80 µg of total protein was incubated with 200 U lambda protein phosphatase (New England Biolabs, Ipswich, MA) and 1 mM MnCl_2_ at 30°C for 30 minutes. Activation of Na_3_VO_4_ was performed according to Gordon [48], and activated Na_3_VO_4_ was added to a final concentration of 20 mM. The reaction was stopped with 5× SDS loading buffer, boiled for 5 min, and loaded onto a 12% SDS-PAGE gel. HRB1∶GFP∶His was detected by western blot with an anti-GFP antibody (Santa Cruz Biotechnology, Santa Cruz, CA).

Agrobacteria carrying pMDC83-control or pMDC83-PP7∶GFP∶His and pBin61-P19 were co-infiltrated into Nicotiana leaves. Approximately 12 g of tissues was harvested and PP7∶GFP∶His was purified through a nickel column as described in the section of *in vivo* affinity-precipitation [34]. The His-tagged PP7∶GFP was then eluted in 200 µl plant protein extraction buffer with 200 mM imidazole and dialyzed against plant protein extraction buffer without imidazole. A total of 10 µg of GFP∶His or PP7∶GFP∶His protein was incubated with 80 µg of total protein prepared from HRB1∶GFP∶His transgenic plants in the presence of 5 mM MnCl_2_ at 30°C for 30 minutes. The subsequent analysis was performed as described above. The D to A mutation at amino acid position 116 in PP7 was converted using the QuikChange™ site-directed mutagenesis kit (Stratagene, San Diego, CA). The primers used were GGAGACTATGTGGCTCGCGGTGCTT and CCAAGCACCGCGAGCCACATAGTCTC.

The phosphorylation status of HRB1∶Myc was also examined in leaves from 3-week-old Col or *pp7* in the dark or under 4.93 µmol/m^2^/s blue light for 2 hours. Protein extracts were prepared in buffer for gel filtration [49] and loaded onto an SDS-PAGE gel. The protein blot was probed with an antibody against Myc tag (Santa Cruz Biotechnology, Santa Cruz, CA).

### Gel filtration, RT–PCR, and phototropic response analysis

The gel filtration profiles for HRB1∶Myc or HRB1∶GFP and PP7∶Myc were analyzed as previously described [49]. Total RNA isolation and RT-PCR analysis were performed as previously described [49]. The hypocotyl phototropic responses of Ws, *hrb1*, Col, and *pp7* were examined according to Inada et al. with some modifications [16]. Four-day-old etiolated seedlings were irradiated for 16 hours with 9.86 µmol/m^2^/s unilateral blue light. The curvatures were measured with image J software.

### Accession numbers

The Sequences of the genes in this paper can be found in the Arabidopsis Genome Initiative database with the following accession numbers: HRB1 (At5G49230), PP7 (At5G63870), Di19 (At1G56280), ACTIN2 (At3G18780) and UBQ10 (At4G05320).

## Supporting Information

Figure S1 Wild type and mutated PP7 proteins accumulate to similar levels in yeast cells. Total proteins were isolated from yeast that carry the GAD, GAD∶PP7, and GAD∶PP7m constructs and separated by SDS-PAGE (upper). The western blot was probed with an anti-HA antibody. Ponceau S staining to show equal protein loading (lower).
(TIF)

Figure S2 A T-DNA insertion line contains a *PP7* knock-down lesion. (A) Schematic diagram showing T-DNA insertion in the *PP7* gene. The filled bars indicate the 5′ and 3′ UTRs, rectangles indicate the exon, and lines indicate the intron. Numbers indicate the beginning and end of each exon. Primers used and their relative positions are shown with arrows. (B) PCR verification of the T-DNA insertion in *pp7*. The full-length PP7 coding region was amplified with primers PP7-5 and PP7-3 from genomic DNA. The T-DNA insertion was genotyped with primers lb5 and PP7-3. (C) RT-PCR analysis of *PP7* expression in Col and *pp7* with two pairs of primers, PP7-5/PP7-851 and PP7-5/PP7-629. *ACTIN* and *UBQ10* were used as controls. (D) Hypocotyl length of 4-day-old Col and *pp7* seedlings in the dark or under 5 µmol/m^2^/s red, 0.05 µmol/m^2^/s far-red, or 1 or 6 µmol/m^2^/s blue light. The hypocotyl length of *pp7* is significantly different from that of Col under 1 or 6 µmol/m^2^/s blue light (n = 50, P<0.0001).
(TIF)

Figure S3 *HRB1* and *PP7* interact to control hypocotyl elongation. Hypocotyl length of 4-day-old Ws, *hrb1*, *PP7 RNAi* in Ws (*RNAi Ws*) or *hrb1* (*RNAi hrb1*) seedlings in the dark and under 4.8 µmol/m^2^/s red or 1 and 5 µmol/m^2^/s blue light. The hypocotyl length of *RNAi hrb1* is significantly different from that of *RNAi Ws* under 5 µmol/m^2^/s blue light (n = 50, P<0.0001).
(TIF)

Figure S4 *pp7* mutation does not affect HRB1 subcellular localization. Subcellular localization of HRB1∶GFP in the guard cells of Col or *pp7* in the dark or under 4.93 µmol/m^2^/s blue light for 2 hours. Propidium iodide (PI) fluorescence shows the cell shape.
(TIF)

Figure S5 HRB1 complex and function are examined in the *cry1 cry2* or *phot1 phot2* double mutants. Gel filtration profiles of HRB1∶Myc from the leaves of 4-week-old *cry1 cry2* (A) or *phot1 phot2* (B) plants in the dark (top) or under 4.93 µmol/m^2^/s blue light for 3 hours (bottom). (C) Stomatal aperture of 4-week-old Col, *35S::HRB1:MYC* in Col (*HRB1OE*), *cry1 cry2*, *35S::HRB1:MYC* in *cry1 cry2* (*HRB1OE crys*), *phot1 phot2*, and *35S::HRB1:MYC* in *phot1 phot2* (*HRB1OE phots*). Significance levels: P<0.01 between a and b; P<0.005 between a and c; P<0.0001 between b and c; P<0.05 between d and e; P<0.0001 between d and f; P<0.0001 between e and f. Plants were in the dark or treated with 0.22 or 1.15 µmol/m^2^/s blue light supplemented with 25 µmol/m^2^/s red light for 2 hours. Data presented are means plus or minus standard errors (n = 50).
(TIF)

Figure S6 *hrb1* has an altered phototropic response. The hypocotyl phototropic responses of 4-day-old etiolated Col, *phot1 phot2*, *pp7*, Ws, and *hrb1* seedlings to either 5 or 10 µmol/m^2^/s unilateral blue light. The numbers after the name of the mutants indicate the independently propagated lines of the same mutant. Experiments were repeated three times, and a representative set of data is shown. The phototropic response of *phot1 phot2* is significantly different from that of Col (n = 50, P<0.0001), and the phototropic response of *hrb1* is significantly different from that of Ws (n = 50, P<0.001).
(TIF)

Figure S7 Blue, red or far-red light does not alter the phosphorylation status of HRB1. Accumulation of HRB1∶Myc in the leaves of 4-week-old transgenic plants in the dark and in response to 5 µmol/m^2^/s blue light, 15 µmol/m^2^/s red light or 1 µmol/m^2^/s far-red light for 2 hours. The blots were stripped and re-probed with an antibody against ß-tubulin.
(TIF)
