# Supplementary figures and images for: Hypersensitive to Red and Blue 1 and Its Modification by Protein Phosphatase 7 Are Implicated in the Control of Arabidopsis Stomatal Aperture
Source: PLoS Genet. 2012 May 10;8(5):e1002674. doi: 10.1371/journal.pgen.1002674 (PMC3349726; doi:10.1371/journal.pgen.1002674)

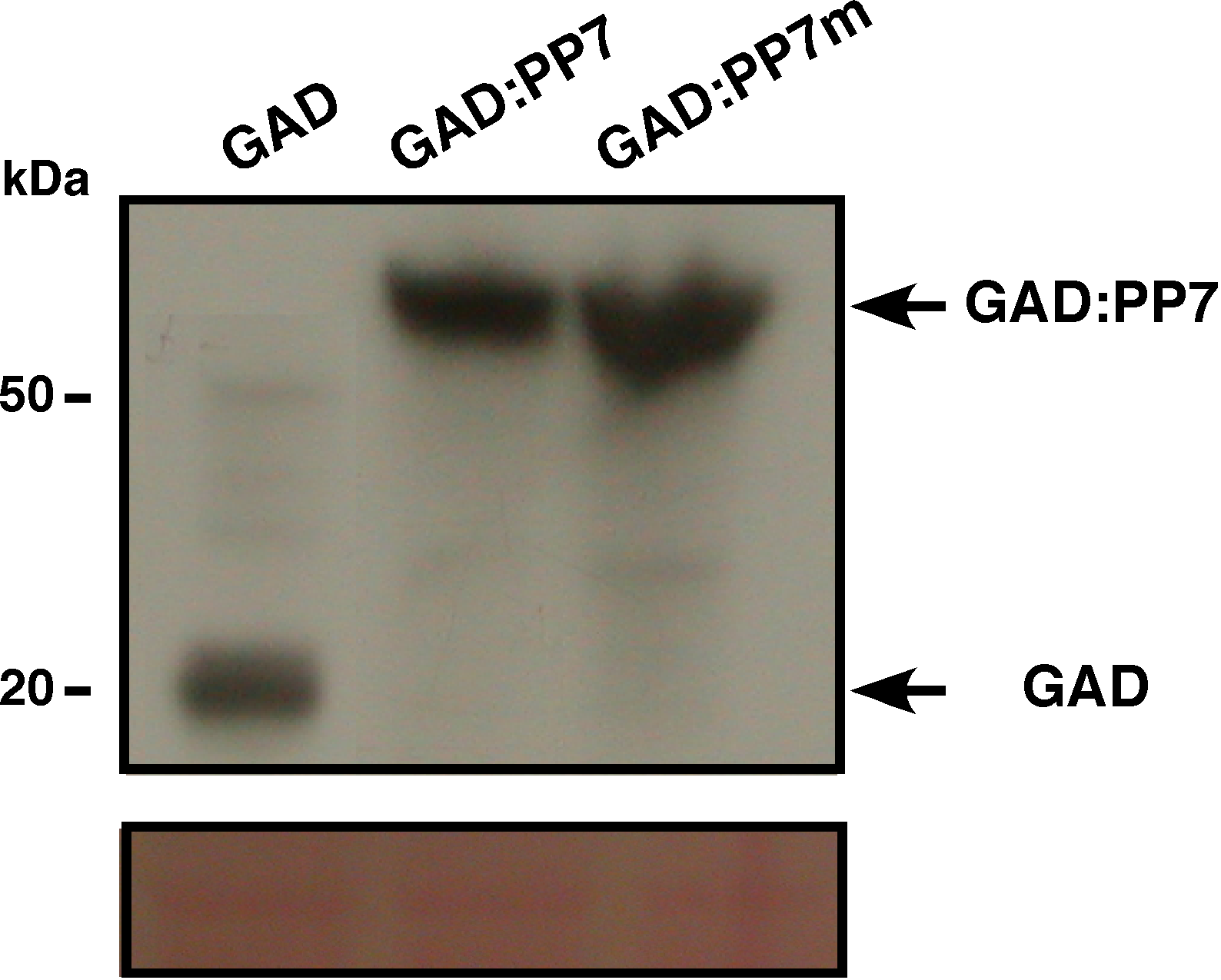

Supplement: Figure S1 — Wild type and mutated PP7 proteins accumulate to similar levels in yeast cells. Total proteins were isolated from yeast that carry the GAD, GAD∶PP7, and GAD∶PP7m constructs and separated by SDS-PAGE (upper). The western blot was probed with an anti-HA antibody. Ponceau S staining to show equal protein loading (lower). (TIF) [file pgen.1002674.s001.tif]

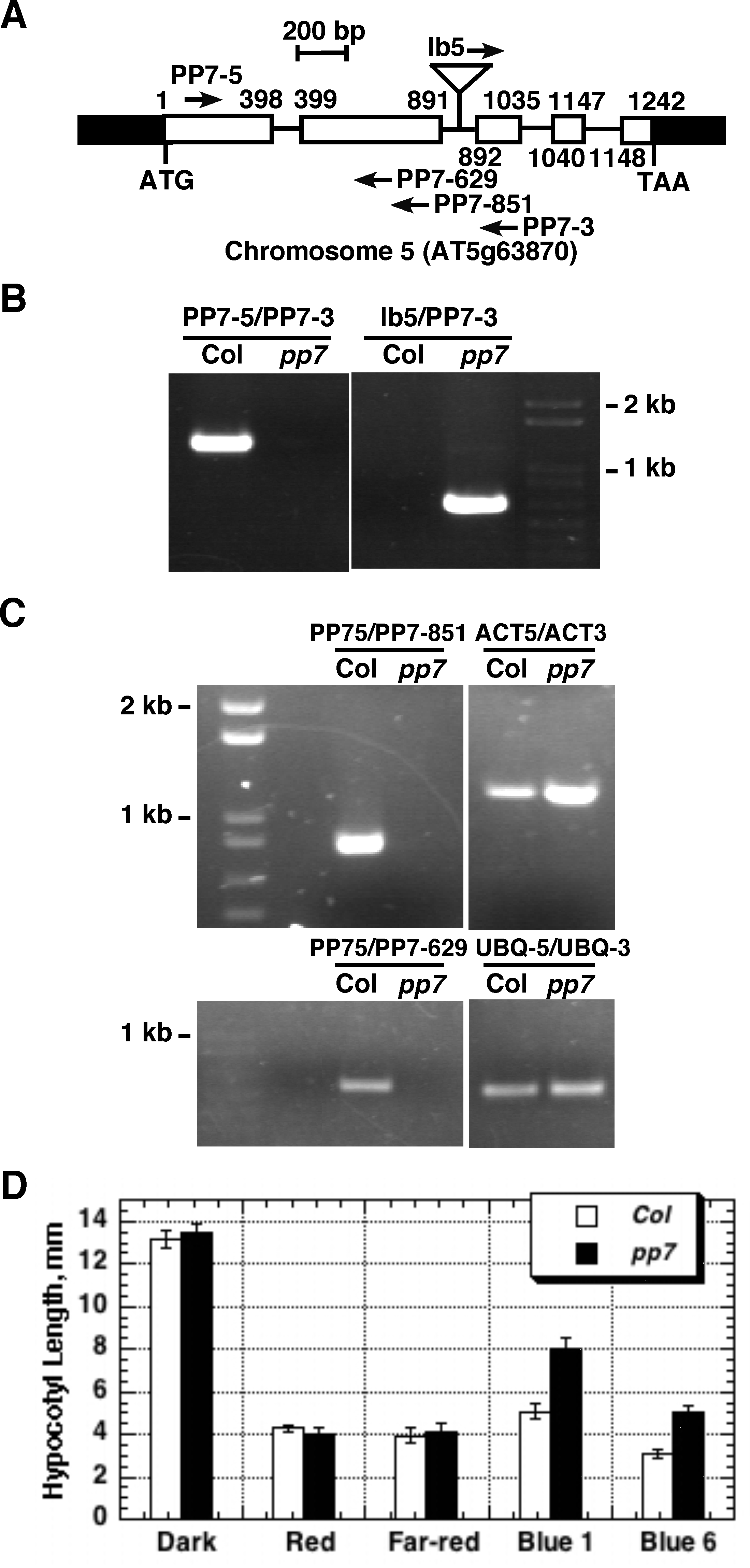

Supplement: Figure S2 — A T-DNA insertion line contains a PP7 knock-down lesion. (A) Schematic diagram showing T-DNA insertion in the PP7 gene. The filled bars indicate the 5′ and 3′ UTRs, rectangles indicate the exon, and lines indicate the intron. Numbers indicate the beginning and end of each exon. Primers used and their relative positions are shown with arrows. (B) PCR verification of the T-DNA insertion in pp7. The full-length PP7 coding region was amplified with primers PP7-5 and PP7-3 from genomic DNA. The T-DNA insertion was genotyped with primers lb5 and PP7-3. (C) RT-PCR analysis of PP7 expression in Col and pp7 with two pairs of primers, PP7-5/PP7-851 and PP7-5/PP7-629. ACTIN and UBQ10 were used as controls. (D) Hypocotyl length of 4-day-old Col and pp7 seedlings in the dark or under 5 µmol/m2/s red, 0.05 µmol/m2/s far-red, or 1 or 6 µmol/m2/s blue light. The hypocotyl length of pp7 is significantly different from that of Col under 1 or 6 µmol/m2/s blue light (n = 50, P<0.0001). (TIF) [file pgen.1002674.s002.tif]

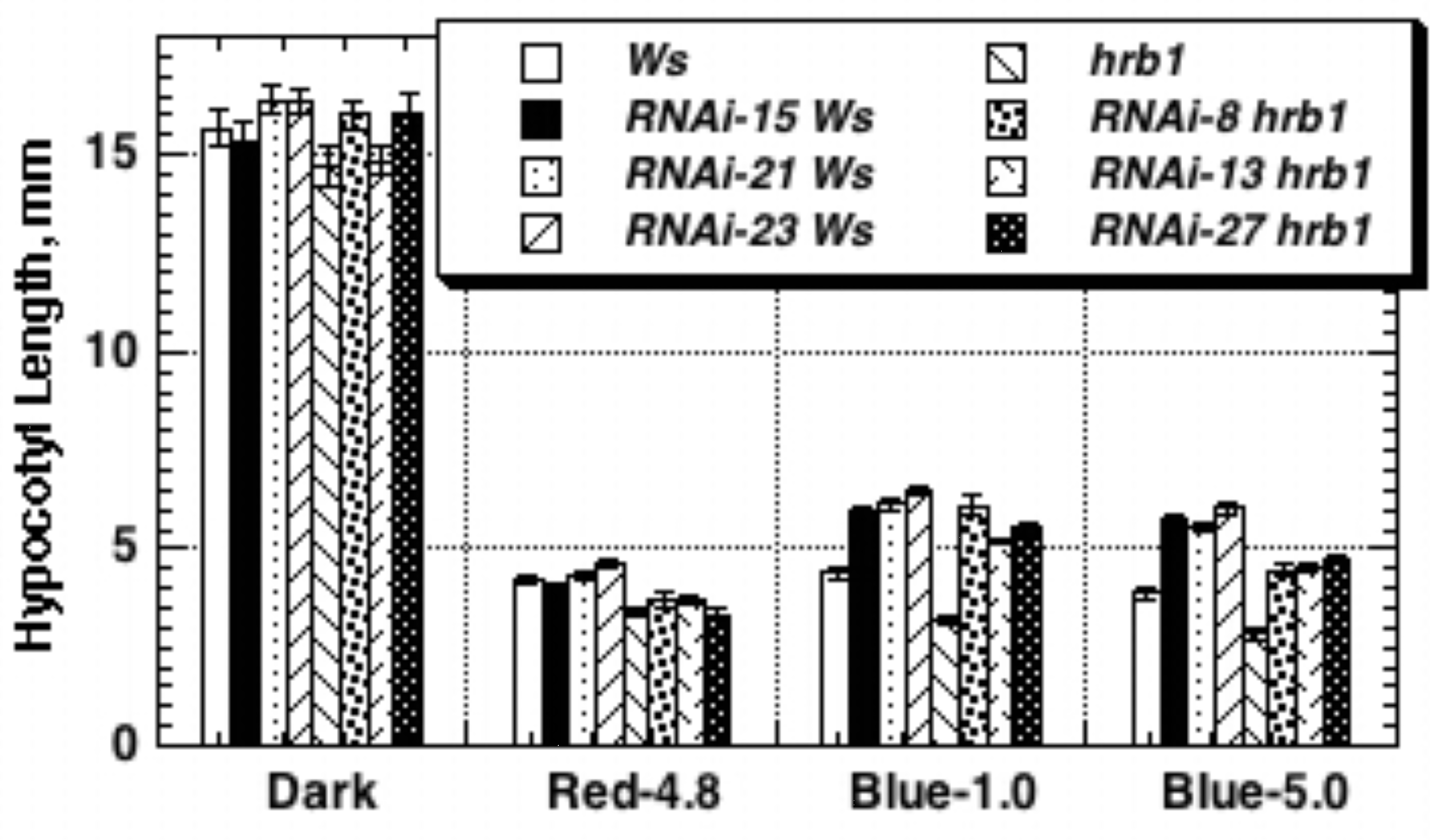

Supplement: Figure S3 — HRB1 and PP7 interact to control hypocotyl elongation. Hypocotyl length of 4-day-old Ws, hrb1, PP7 RNAi in Ws (RNAi Ws) or hrb1 (RNAi hrb1) seedlings in the dark and under 4.8 µmol/m2/s red or 1 and 5 µmol/m2/s blue light. The hypocotyl length of RNAi hrb1 is significantly different from that of RNAi Ws under 5 µmol/m2/s blue light (n = 50, P<0.0001). (TIF) [file pgen.1002674.s003.tif]

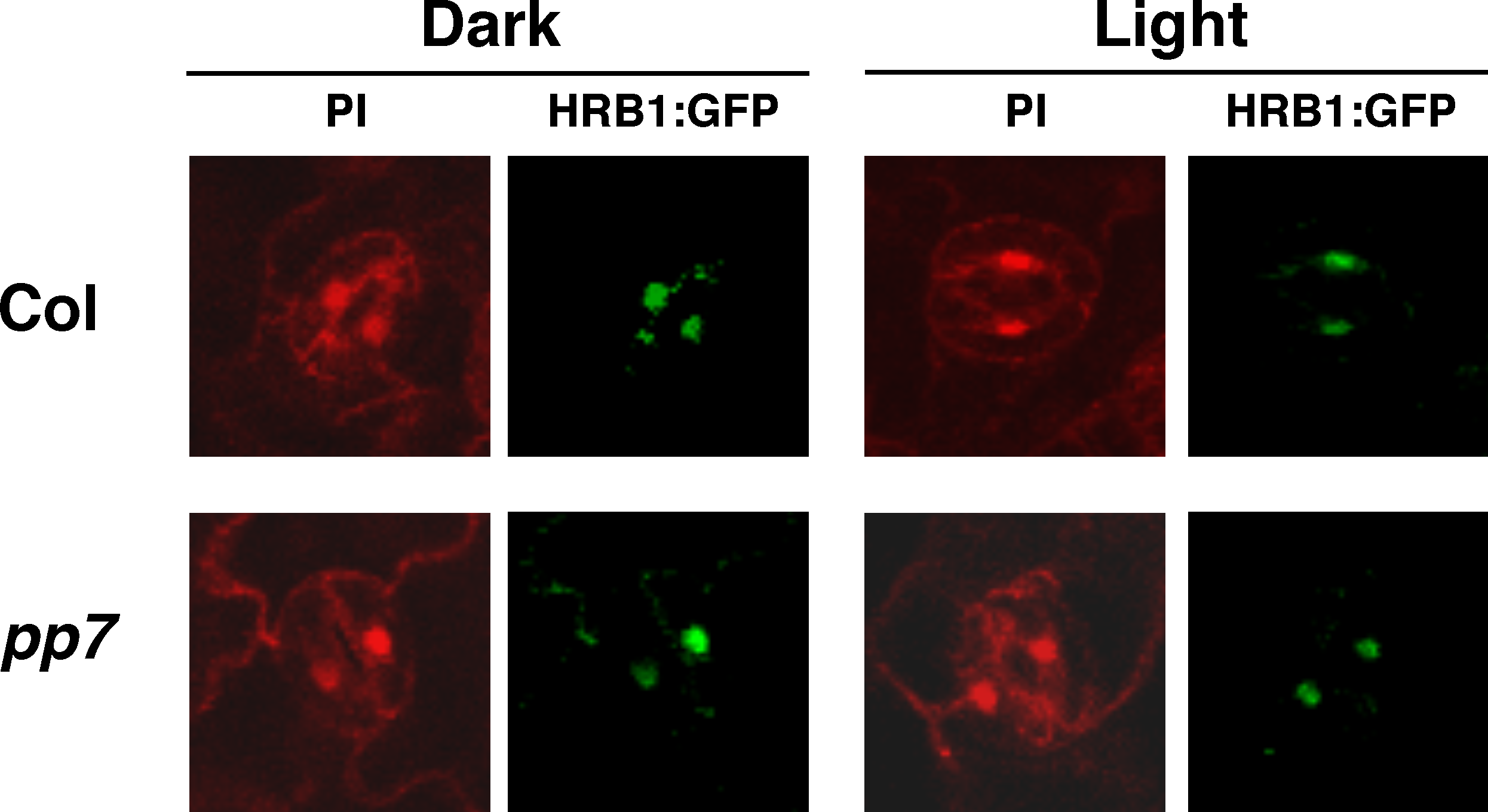

Supplement: Figure S4 — pp7 mutation does not affect HRB1 subcellular localization. Subcellular localization of HRB1∶GFP in the guard cells of Col or pp7 in the dark or under 4.93 µmol/m2/s blue light for 2 hours. Propidium iodide (PI) fluorescence shows the cell shape. (TIF) [file pgen.1002674.s004.tif]

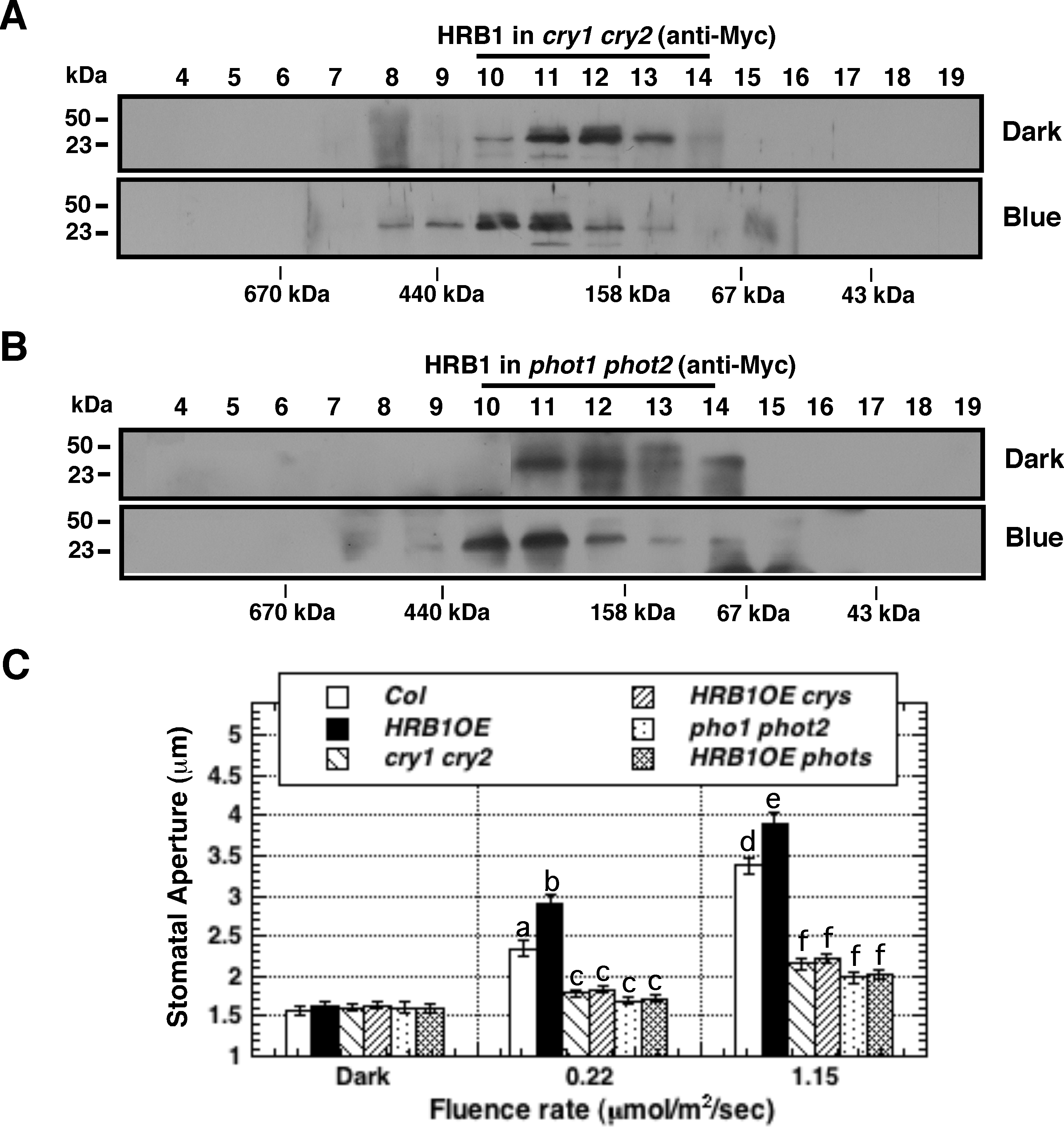

Supplement: Figure S5 — HRB1 complex and function are examined in the cry1 cry2 or phot1 phot2 double mutants. Gel filtration profiles of HRB1∶Myc from the leaves of 4-week-old cry1 cry2 (A) or phot1 phot2 (B) plants in the dark (top) or under 4.93 µmol/m2/s blue light for 3 hours (bottom). (C) Stomatal aperture of 4-week-old Col, 35S::HRB1:MYC in Col (HRB1OE), cry1 cry2, 35S::HRB1:MYC in cry1 cry2 (HRB1OE crys), phot1 phot2, and 35S::HRB1:MYC in phot1 phot2 (HRB1OE phots). Significance levels: P<0.01 between a and b; P<0.005 between a and c; P<0.0001 between b and c; P<0.05 between d and e; P<0.0001 between d and f; P<0.0001 between e and f. Plants were in the dark or treated with 0.22 or 1.15 µmol/m2/s blue light supplemented with 25 µmol/m2/s red light for 2 hours. Data presented are means plus or minus standard errors (n = 50). (TIF) [file pgen.1002674.s005.tif]

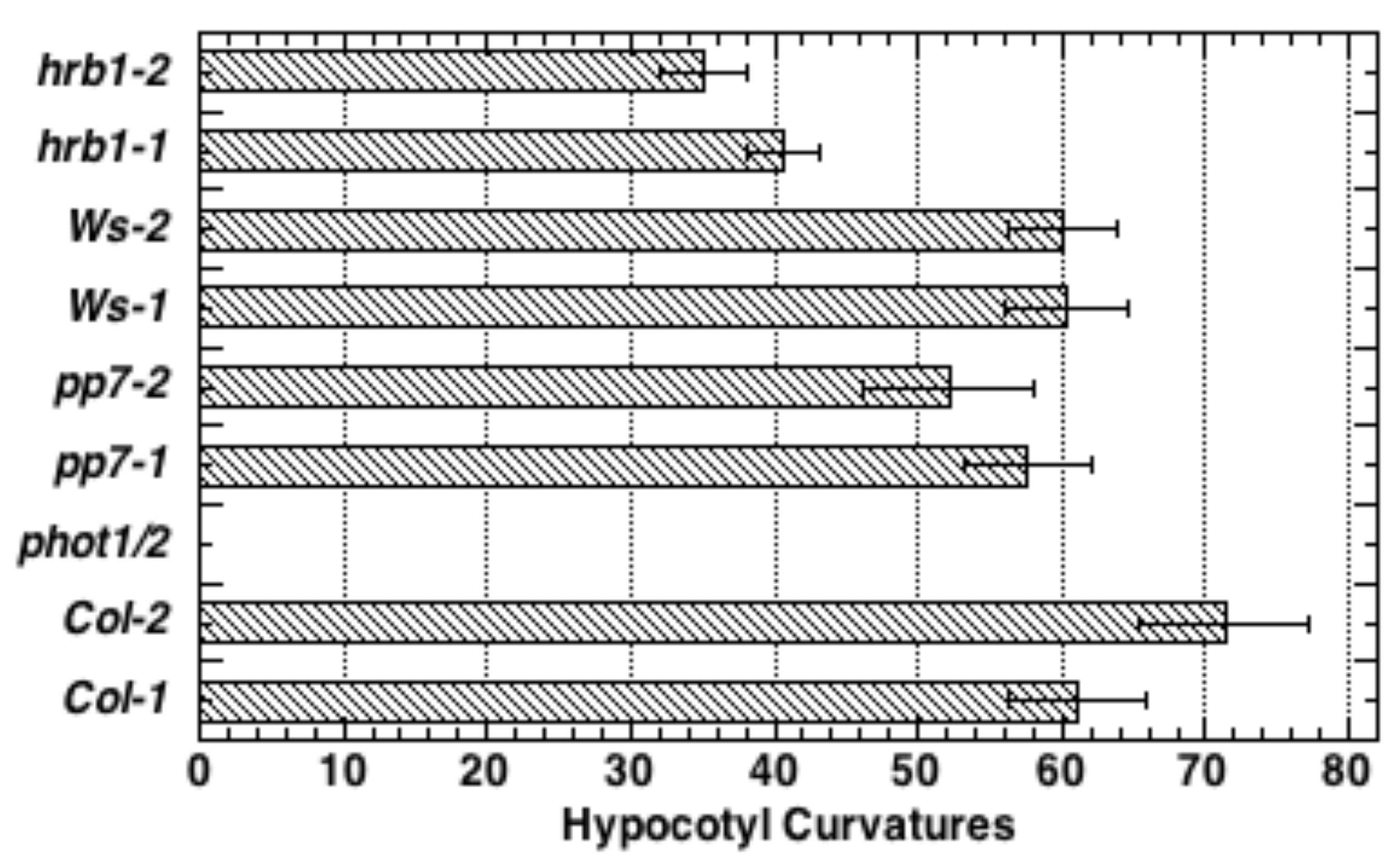

Supplement: Figure S6 — hrb1 has an altered phototropic response. The hypocotyl phototropic responses of 4-day-old etiolated Col, phot1 phot2, pp7, Ws, and hrb1 seedlings to either 5 or 10 µmol/m2/s unilateral blue light. The numbers after the name of the mutants indicate the independently propagated lines of the same mutant. Experiments were repeated three times, and a representative set of data is shown. The phototropic response of phot1 phot2 is significantly different from that of Col (n = 50, P<0.0001), and the phototropic response of hrb1 is significantly different from that of Ws (n = 50, P<0.001). (TIF) [file pgen.1002674.s006.tif]

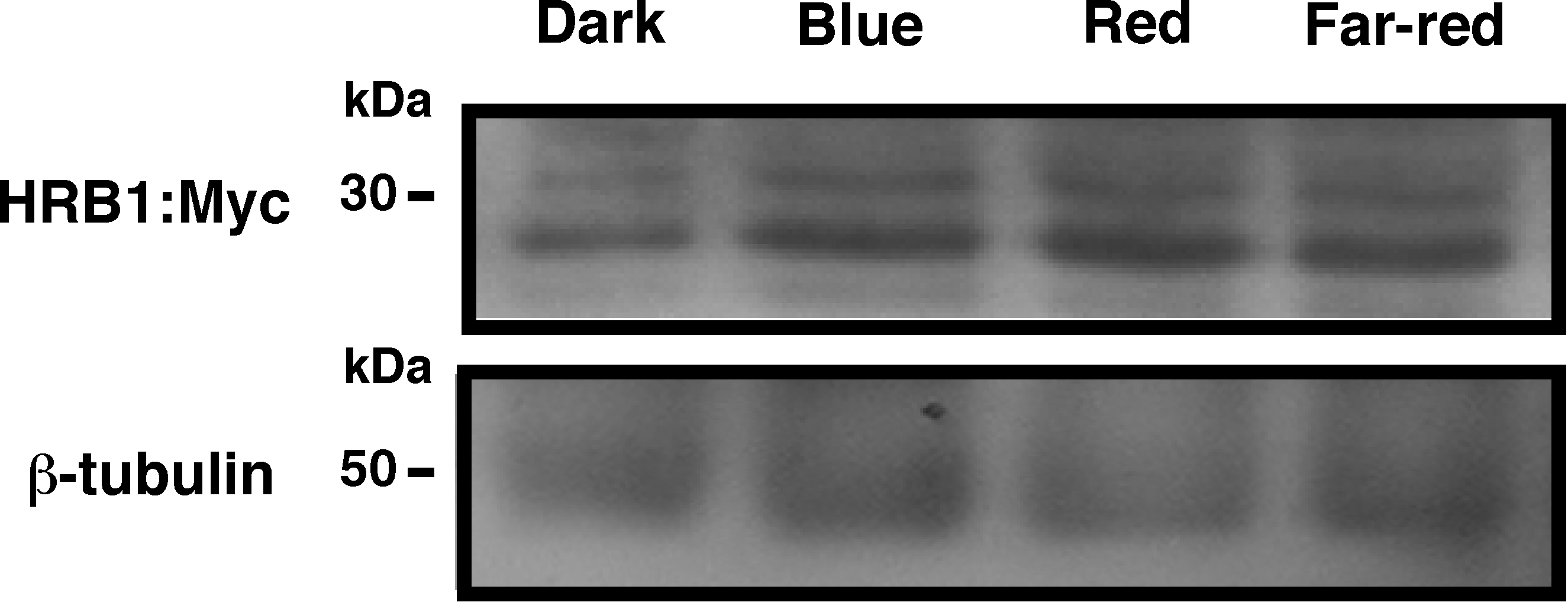

Supplement: Figure S7 — Blue, red or far-red light does not alter the phosphorylation status of HRB1. Accumulation of HRB1∶Myc in the leaves of 4-week-old transgenic plants in the dark and in response to 5 µmol/m2/s blue light, 15 µmol/m2/s red light or 1 µmol/m2/s far-red light for 2 hours. The blots were stripped and re-probed with an antibody against ß-tubulin. (TIF) [file pgen.1002674.s007.tif]
